# 

**Published:** 1871-07

**Authors:** 


					﻿Vol. XII.
JULY, 1871.
No. 7.
CHICAGO
Medical Examiner,
N. S. DAVIS, M.D., Editor,
AND
F. II. Davis, M.D., Assist.-Editoi
ri'JHIS. S3.OO I’l l I? AIXIXTAT, I TV ADVANCE.
CHICAGO:
ROBERT FERGUS’ SONS, PRINTERS,
12 CLARK AND 242—248 ILLINOIS STREET.
1871.
				

